# Clinical Characteristics of Acute Kidney Injury Associated with Tropical Acute Febrile Illness

**DOI:** 10.3390/tropicalmed8030147

**Published:** 2023-02-27

**Authors:** Fardosa Dahir Omar, Weerapong Phumratanaprapin, Udomsak Silachamroon, Borimas Hanboonkunupakarn, Natthida Sriboonvorakul, Janjira Thaipadungpanit, Wirichada Pan-ngum

**Affiliations:** 1 Department of Clinical Tropical Medicine, Faculty of Tropical Medicine, Mahidol University, Bangkok 10400, Thailand; 2 Faculty of Medicine and Health Sciences, SIMAD University, Mogadishu 2526, Somalia; 3 Mahidol-Oxford Research Unit (MORU), Faculty of Tropical Medicine, Mahidol University, Bangkok 10400, Thailand; 4 Department of Tropical Hygiene, Faculty of Tropical Medicine, Mahidol University, Bangkok 10400, Thailand

**Keywords:** acute kidney injury, acute febrile illness, tropical diseases, prevalence, clinical, outcome

## Abstract

Tropical acute febrile illness (TAFI) is one of the most frequent causes of acute kidney injury (AKI). The prevalence of AKI varies worldwide because there are limited reports available and different definitions are used. This retrospective study aimed to determine the prevalence, clinical characteristics, and outcomes of AKI associated with TAFI among patients. Patients with TAFI were classified into non-AKI and AKI cases based on the Kidney Disease Improving Global Outcomes (KDIGO) criteria. Of 1019 patients with TAFI, 69 cases were classified as having AKI, a prevalence of 6.8%. Signs, symptoms, and laboratory results were significantly abnormal in the AKI group, including high-grade fever, dyspnea, leukocytosis, severe transaminitis, hypoalbuminemia, metabolic acidosis, and proteinuria. 20.3% of AKI cases required dialysis and 18.8% received inotropic drugs. Seven patients died, all of which were in the AKI group. Risk factors for TAFI-associated AKI were being male (adjusted odds ratio (AOR) 3.1; 95% CI 1.3–7.4), respiratory failure (AOR 4.6 95% CI 1.5–14.1), hyperbilirubinemia (AOR 2.4; 95% CI 1.1–4.9), and obesity (AOR 2.9; 95% CI 1.4–6). We recommend clinicians investigate kidney function in patients with TAFI who have these risk factors to detect AKI in its early stages and offer appropriate management.

## 1. Introduction

Fever, or acute febrile illness (AFI), is defined as an increase in body temperature caused by alterations in the hypothalamic thermoregulatory center [1]. There is a wide range of AFIs with different etiologies and induced by local outbreaks of disease, which can vary from region to region and country to country [2]. Tropical acute febrile illness (TAFI), which occurs in individuals living in tropical and subtropical regions, is defined as an AFI with a fever of duration less than 14 days. TAFI has no specific clinical signs and symptoms; most patients complain of fever, myalgia, arthralgia, vomiting, breathlessness, cough, chest pain, headache, rash, conjunctival congestion, or other symptoms. There are many infections that manifest as AFI in tropical countries, including dengue fever, typhoid fever, leptospirosis, rickettsia, influenza, and malaria [3,4]. In some AFI cases, patients also develop acute kidney injury (AKI). AKI is characterized by a rapid loss of the kidney’s excretory function, with or without oliguria, which commonly occurs over the course of hours to days. AKI is common in hospitalized patients, especially critically ill patients [5]. 

The pathophysiology of AKI in TAFI remains unclear. It is likely multifactorial and may differ based on infectious etiologies and their clinical presentation. Different hypotheses have been proposed, including direct injury to kidney tissue, immune mechanisms, hemolysis, cytoadherence of parasite-infected erythrocytes, intravascular coagulation, severe hyperpyrexia, vasculitis, and a secondary outcome of rhabdomyolysis [6,7,8,9,10,11,12,13,14,15,16,17,18,19,20]. In 2016, the incidence of AKI in Asia was as follows: 31% in Southeastern Asia, 19.4% in Eastern Asia, 16.7% in Western Asia, 9% in Central Asia, and 7.5% in Southern Asia [21]. The reported incidence of AKI varied based on the definition of AKI used and by ethnicity [22]. Mortality in AKI remains high globally, even in high-resource settings [23]. Poor health awareness and a lack of good diagnostic tests, limited resources, and poor sanitation are the major reasons for the differences in AKI outcomes between developing and developed countries [23]. AKI in the tropics is little known due to limited reporting and differences in the definitions of AKI used. Early diagnosis is essential to prevent AKI in patients with TAFI, which can be a common outcome of many infections, such as dengue, malaria, influenza, rickettsia, and leptospirosis. The present study aimed to determine the prevalence, clinical characteristics, and outcomes of AKI associated with tropical acute febrile illness.

## 2. Materials and Methods

### 2.1. Study Design

This retrospective study received ethical approval from the Ethics Committee of the Faculty of Tropical Medicine, Mahidol University, Thailand (Certificate No. MUTM 2019–018-01). The Ethics Committee waived the requirement for informed consent and the data were fully anonymized before analysis. We assessed the medical records of patients with TAFI who were admitted to the Hospital for Tropical Diseases, Faculty of Tropical Medicine, Mahidol University, Thailand, between January 2015 and December 2018. The international statistical classification of diseases and related health problems, 10th revision (ICD10) criteria were used to search for medical records of patients diagnosed with TAFI. The inclusion criteria were TAFI patients aged ≥ 18 years who had a history of fever < 2 weeks and a fever ≥ 37.5 °C during their first 24-h period of hospitalization. Patients with no serum creatinine result during hospitalization, missing clinical data in their medical records, non-specific or non-infectious causes of AFI, specific organ involvement with non-tropical diseases, or a fever of unknown origin were excluded from the study. The medical records of included cases were classified into AKI and non-AKI groups, based on the Kidney Disease Improving Global Outcomes (KDIGO) criteria [24]. A diagnosis of AKI was based solely on serum creatinine (SCr) results. Patient data, including both hard copies and electronic records, were reviewed on a case-by-case basis. Demographic, clinical, and laboratory data of eligible patients were obtained. Demographic details and clinical presentations were recorded upon hospital admission, while laboratory data were collected during a patient’s hospitalization.

### 2.2. Clinical Definitions

AKI is a complex clinical disorder characterized by a rapid loss in the kidney’s excretory function, with or without oliguria, occuring over the course of a few hours to days. It is closely associated with severe morbidity and mortality [5]. According to the KDIGO criteria, AKI is defined as an increase in SCr of ≥0.3 mg/dL within 48 h or an increase of ≥1.5 times the baseline within 7 days. It is divided into: stage 1, SCr increase >0.3 mg/dL or SCr increase 1.5–1.9-times the baseline; stage 2, SCr increase 2–2.9-times the baseline; stage 3, SCr increase 3-times the baseline, or the initiation of renal replacement therapy (RRT) [25,26]. Patients’ SCr results on admission were used as the baseline for comparison of their kidney function during hospitalization. For patients who had a single SCr result, the baseline SCr was estimated using the Chronic Kidney Disease Epidemiology Collaboration (CKD-EPI) equation [27]. Other AKI criteria, including conventional, RIFLE and AKIN, were defined (see Supplementary File S1) and explored in this study.

In this study, patients were observed for the presence of clinical conditions and their severity grading. These included hyperbilirubinemia, transaminitis, metabolic acidosis, proteinuria, hematuria, pyuria, severe thrombocytopenia, respiratory failure, multi-organ dysfunction (MOD), and obesity. Further details on the definition of these conditions can be found in Supplementary File S1.

### 2.3. Statistical Analysis

The sample size required was 1013 patients, based on the estimated proportion of patients with TAFI of 0.54 (Nair et al., 2016), with a margin of error of 0.025 and alpha 0.05 [28,29]. The statistical analysis was performed using SPSS software (version 18) Chicago: SPSS Inc. (Chicago, IL, USA) Quantitative variables are given as medians with interquartile ranges (IQRs). For hypothesis testing, appropriate tests, including the Student’s *t*-test or the Mann-Whitney U test, were selected, depending on the data distribution. All categorical variables are presented as numbers and percentages. The chi-square test and Fisher’s exact test were used for group comparisons based on cell values in the tables. A *p*-value of 0.05 or less was considered to indicate statistical significance. The Receiver Operating Characteristic (ROC) curve was applied to evaluate the diagnostic ability of tests. The optimal cut-off value of a test was obtained by maximizing the area under the ROC curve, at which the sensitivity and specificity were then reported.

## 3. Results

**Figure 1:**
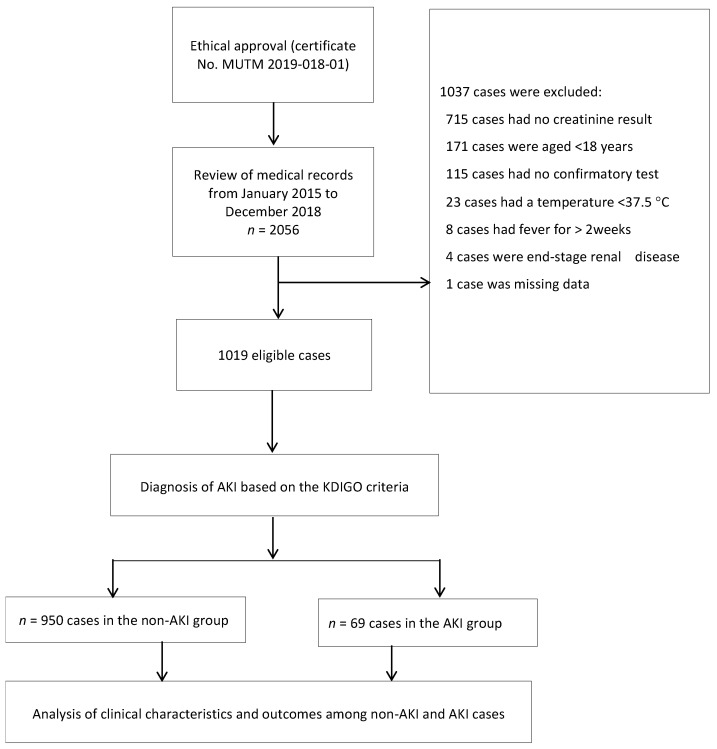
Study flow-diagram.

Overall, a total of 2056 medical records of patients diagnosed with TAFI were screened for the study, of which 1037 cases were excluded, leaving 1019 patients who were eligible for the study, as shown in Figure 1. Of the eligible cases, 603 (59%) had just one SCr result. Therefore, their baseline SCr was estimated using the CKD-EPI equation.

### 3.1. Characteristics of Eligible Cases and Prevalence of AKI in Patients with TAFI

In total, 950 cases were classified into the non-AKI group, with the remaining 69 cases classified into the AKI group. Of the latter cases, 41/69 (59.4%) had dengue, 14/69 (20.3%) had malaria, 11/69 (15.9%) had influenza, 2/69 (2.9%) had rickettsial illness (murine typhus), and 1/69 (1.4%) had leptospirosis; there were no patients with mixed infections (Table 1). The prevalence of AKI by KDIGO criteria, AKIN criteria, RIFLE criteria, and conventional criteria was 6.8%, 5.9%, 4.1%, and 4.0%, respectively. The proportion of AKI by KDIGO criteria in stages 1, 2, and 3 was 45/69 (65.2%), 8/69 (11.6%), and 16/69 (23.2%), respectively (Table 2). In comparison with the non-AKI group, the AKI group comprised mostly males (50/69, 72.5%, *p* = 0.004), with a female to male ratio of 1:2.6. The distribution of AKI by TAFI is shown in Table 1. Among dengue infections, 41/767 (5.3%) had AKI; among malaria infections, 14/131 (10.7%) had AKI; among influenza infections, 11/106 (10.4%) had AKI; among rickettsial infections, 2/11 (18.2%) had AKI; and 1/2 (50%) of leptospirosis infections had AKI. According to the WHO 2009 case definition for dengue, 15/48 (31.3%) of severe dengue cases were in the AKI group. There were 131 malaria cases, of which 14 (10.7%) were in the AKI group: 10 with Plasmodium falciparum (Pf) malaria and 4 with Plasmodium vivax (Pv) malaria. Of the 11 (10.4%) influenza cases in the AKI group, 9 had influenza A and 2 had influenza B. Lastly, 2/2 (100%) of rickettsial cases in the AKI group had murine typhus.

The clinical characteristics of the patients with TAFI in the AKI and non-AKI groups are summarized in Table 3. All, except two, patients complained of fever on admission. Chills and headache were reported among half of all patients in both groups. Nausea/vomiting and other symptoms, including abdominal pain and cough, were around 30% in both groups. Arthralgia was rarely detected, at less than 5% in each group. Pallor and dyspnea were significantly more common among AKI cases than non-AKI cases, at 10.1% and 14.5%, respectively. Myalgia was significantly lower in the AKI compared with the non-AKI group, i.e., 45% vs. 66%, respectively. The major underlying diseases in patients with AKI were diabetes mellitus (11/69, 15.9%), chronic kidney disease (6/69, 8.7%), hepatitis B virus (4/69, 5.8%), human immunodeficiency virus (5/69, 7.2%), and asthma (6/69, 8.7%), with statistically significant differences between the AKI and non-AKI groups. Hypertension (8/69, 11.6%) and dyslipidemia (21/69, 30.4%) showed no statistically significant differences between the AKI and non-AKI groups.

Laboratory test results from patient samples collected on admission are shown in Table 4. Statistically significant differences were observed between the AKI and non-AKI groups in serum sodium, serum potassium, serum bicarbonate, serum total bilirubin, urine specific gravity, WBCs, neutrophils, lymphocytes, atypical lymphocytes, RBCs, and AST. In the AKI group, 8/69 (11.6%) cases had serum creatinine > 3.0 mg/dL and presented with AKI stage 3 on admission. In AKI cases, 31/58 (53.4%) developed hyperbilirubinemia (TB > 1.2 mg/dL) on admission. There were 22/56 (39.3%) AKI cases with moderate to severe transaminitis. The median specific gravity of urine from patients in the AKI group was 1.020 (1.01–1.025; *p* < 0.001), which was significantly higher than in the non-AKI group. Proteinuria was significantly higher in the AKI group (27/53, 50.9%, *p* < 0.001). Urine sedimentation was higher in the AKI group, including hematuria (11/53, 20.8%) and pyuria (8/53, 15.1%), but this was not statistically significant (*p* > 0.05).

### 3.2. Complications and Outcomes of AKI in Patients with TAFI

Complications present in cases of AKI included severe transaminitis (13/56, 23.2%) and hypoalbuminemia (21/58, 36.2%), which were significantly higher than in the non-AKI group (*p* < 0.05). Metabolic acidosis was present in 10/69 (14.5%) cases; 23/69 (33.3%) cases developed respiratory failure and required mechanical ventilation; 20/69 (28.9%) patients were admitted to the intensive care unit (ICU); 13/69 (18.8%) patients developed multi-organ dysfunction, and 6/69 (8.7%) had underlying CKD. All were significantly higher than in the non-AKI groups. Only severe thrombocytopenia (29/69, 42%) showed no significant difference. There were 60/69 (86.9%) patients with AKI who improved and recovered, while one patient did not improve. Another case was transferred to lung cancer treatment. There were 13/69 (18.8%) and 14/69 (20.3%) patients who received inotropic drugs (norepinephrine) and hemodialysis, respectively. Among patients with hemodialysis, 4/14 (28.6%) patients were on intermittent hemodialysis, 3/14 (21.4%) patients on sustained low-efficiency dialysis (SLED), and 7/14 (50%) patients on continuous renal replacement therapy (CRRT). There were 7/69 (10.1%) patients who died; all were in the AKI group. Most patients who died (5/7, 71.4%) were female. As shown in Table 5, 6/7 (85.7%) patients developed multi-organ dysfunction and all patients developed respiratory failure. All patients who died were classified as having severe dengue with AKI stage 3. The parameters with 5% occurrence of cases and non-complete separation (perfect predictor) were further processed to the risk association analysis.

### 3.3. Factors Associated with AKI

Univariate and multivariate analyses were performed to identify independent risk factors for AKI, quantified by the adjusted odds ratio (AOR), with 95% CI. Categorical variables were assigned to the model, with entry at 0.05 and removal at 0.10, and were scored using “no” as the reference category. We found that male sex (AOR 3.1; 95% CI 1.3–7.4), respiratory failure (AOR 4.6; 95% CI 1.5–14.1), hyperbilirubinemia (AOR 2.4; 95% CI 1.1–4.9), and obesity (AOR 2.9; 95% CI 1.4–6) were risk factors associated with AKI (Table 6).

Based on the common model discrimination method using the receiver operating characteristic curve (ROC), the optimal sensitivity and specificity obtained at the maximum area under the curve of 0.767 (95% CI 0.69–0.85) were 70% and 72%, respectively. 

## 4. Discussion

This retrospective study assessed the prevalence and clinical characteristics of AKI in patients with TAFI. We observed a prevalence of AKI by KDIGO, AKIN, RIFLE, and conventional definitions of 6.8%, 5.9%, 4.1%, and 4.0%, respectively. Three studies of TAFI-associated AKI conducted in India between 2010 and 2018 reported a prevalence between 28–54% using either RIFLE or KDIGO criteria [4,30,31]. One study from Malaysia in 2017 reported TAFI-associated AKI with a prevalence of 41.1%, using KDIGO [32]. The wide range of reported AKI prevalence was due to the different AKI criteria being used, as well as the different etiology of AKI in these places. For the present study, we found six patients with underlying chronic kidney disease (CKD). There were seven underlying CKD cases in our study. However, there remained the possibility of other cases of undiagnosed CKD. CKD has previously been reported as a risk factor for developing AKI [33]. Similarly, many previous studies reported some underlying CKD cases which subsequently developed AKI [30,34]. In our study, we excluded approximately 34.8% (715/2056) of the total screened medical records due to the absence of a creatinine investigation. The clinicians did not request SCr investigation in these patients because they did not have symptoms and signs of AKI, and the clinicians did not suspect AKI. A previous study experienced a similar rate of exclusion (810/2476, 32.7%) during screening due to an absence of SCr results [34]. The infections present in patients with TAFI-associated AKI in our study were dengue (59.4%), malaria (20.3%), influenza (15.9%), rickettsia (2.9%), and leptospirosis (1.5%). The mechanisms of TAFI-associated AKI are complex. The alteration of kidney tubular function resulting from hemodynamic instability and hypotension, cytokine production, and immune complex deposition has been noted for the three major TAFI listed (dengue, malaria, and influenza) in this study [35,36,37]. Dengue shock syndrome (DSS) also increases the risk of AKI [35]. Hemodynamic derangement from parasitized red blood cells and platelets resulting in microvascular blockage (sequestration) is also a major mechanism in malarial AKI [38]. Note that in our study dengue was the largest TAFI group at 75.3% (767/1019). Different studies have reported a high proportion of leptospirosis [4], scrub typhus, malaria, and HIV [30,39,40] among TAFI-associated AKI patients. The causes of TAFI associated with AKI were different from region to region or country to country due to infection cause and population demographics [22,30,41]. In our study, the proportion of patients with AKI in stages 1, 2, and 3 were 65.2%, 11.6%, and 23.2%, respectively. All cases of stage 3 AKI in our study were associated with severe dengue infection or falciparum malaria.

Most of the patients with stage 1 AKI were diagnosed by laboratory investigations [4]; all deaths were in stage 3 AKI. A high-grade fever (>39 °C) was observed in 29% of cases in the AKI group, which was significantly higher than the proportion of patients with high-grade fever in the non-AKI group. Pyrexia causes dehydration and is characteristic of AFI [4]. On admission, a significantly higher proportion of patients with dyspnea (14.5%) and pallor (10.1%) were detected in the AKI group in our study. Previous studies have reported that 20% of AKI cases presenting with dyspnea, metabolic acidosis, and shock are significantly likely to occur with dengue-associated AKI [4,10,34]. There were 50.9% of AKI cases with proteinuria, which was significantly higher than in the non-AKI group, while 20.8% and 15.1% of AKI cases had hematuria and pyuria, respectively, which was not significantly different compared with the non-AKI group. Proteinuria, hematuria, and pyuria have been reported to be associated with AKI elsewhere [42].

The median WBC increased significantly in the AKI group compared to the non-AKI group. Both leukocytosis and leukopenia can increase the risk of developing AKI in critical patients in the ICU [43]. Inflammation plays an important role in tubular cell damage during AKI, with neutrophils being an obvious factor in the inflammatory cascade [44,45]. Lymphocytes play a critical role in the immune inflammatory response, and the production of cytokines participates in the AKI process [46]. We also observed severe transaminitis (23.2%), hypoalbuminemia (36.2%), and hyperbilirubinemia (53.4%) to be higher in the AKI group than in the non-AKI group. Patients who exhibited an increase in transaminitis, hyperbilirubinemia, and hypoalbuminemia were shown in a previous study to be more likely to develop AKI [47]. Hypoalbuminemia is a risk factor for AKI [48]. In this study, the AKI group had a higher proportion of hyponatremia (53.8%) and metabolic acidosis (14.5%) than the non-AKI group. A decline in GFR will cause the retention of waste products, aggravating electrolyte imbalance, including hyponatremia, hyperkalemia, and metabolic acidosis. Metabolic acidosis has been reported to be associated with AKI [49,50,51]. In our study, most of the patients in both the non-AKI and AKI groups recovered and had improved by the time they were discharged. However, all of the deaths we recorded occurred in cases of AKI (10.1%). The etiology of their deaths was unknown, as no autopsies were performed. Among the deaths, 5/7 cases (71.4%) were female, while all patients who died developed respiratory failure, and 6/7 cases (85.7%) had multi-organ dysfunction. All these cases had severe dengue infection and stage 3 AKI. Among the death cases in our study, 5 out of 7 death cases were referred from another hospital with severe dengue, multi-organ dysfunction, and shock on presentation, and 4 out of 5 referred cases were female. In our study, the mortality rate in patients with TAFI was 7/1019 (0.69%). Other studies have reported mortality rates in all cases of AFI, irrespective of AKI, of 3% and 12.3% [4,30]. The variation in mortality rates observed in those studies could be explained by differences in the study populations, rates of ICU admission, underlying diseases, and the different criteria used for the classification of AKI in different studies. However, patients with AKI had significantly higher in-hospital mortality than non-AKI cases, regardless of the AKI definition used [33]. Mortality in the AKI group (10.1%) in our study was significantly lower than the study of acute kidney injury in a tropical country by Daher et al., which reported a mortality of 62.8% [39]. The low mortality rate of our study can be explained by the fact that it was conducted in a tertiary care hospital for tropical diseases in Thailand, where the medical care and services include high-quality ICU settings, the availability of extensive laboratory investigations, mechanical ventilator support, and renal replacement therapy.

In the present study, 18.5% and 20.3% of AKI cases required inotropic drugs and dialysis, respectively. Among the dialysis cases, 21.4% and 50% of them were on SLED and CRRT mode, respectively, according to the hemodynamically unstable conditions. There were no cases in the non-AKI group that required inotropic drugs or dialysis. Previous studies have reported between 7.9% and 44.8% of patients with TAFI-associated AKI requiring dialysis [4,30,39,40,52,53]. In our study, patients in the AKI group had significantly longer hospital stays than patients in the non-AKI group. A similar result was observed in a previous study [34]. 

Similar to some previous studies, males showed a higher risk of developing AKI than females (AOR 3.1; 95% CI 1.3–7.4) [54,55]. Many studies have shown that endoplasmic reticulum stress participates in the development of AKI in both animals and humans, and that the kidney of the male is more vulnerable to endoplasmic reticulum stress [56,57]. Additionally, testosterone has apoptotic and fibrotic effects that are aggravated by the release of TNF-α, and the generation of inflammation leading to AKI. Respiratory failure was shown to be another risk factor for AKI in the present study (AOR 4.6; 95% CI 1.5–14.1). This same result has been noted previously [58]. The pathophysiology of AKI in respiratory failure is not completely understood, but studies have reported many mechanisms that participate in the aggravation of AKI during respiratory failure. For example, increasing intrathoracic pressures with poorly compliant lungs can reduce cardiac output, resulting in inadequate renal perfusion that aggravates AKI [59,60,61]. Hyperbilirubinemia was shown to be one of the risk factors for AKI in the present study (AOR 2.4; 95% CI 1.1–4.9), which agrees with a previous report [62]. There has also been a report of severe hyperbilirubinemia and association of severe AKI in patients with cardiac surgery [63]. Bilirubin and bile salt cause direct damage to the tubular epithelium of the kidney [64]. Furthermore, high bilirubin levels would stimulate renal ischemic-reperfusion injury [65]. Obesity is another risk factor for AKI identified in the present study (AOR 2.9; 95% CI 1.4–6); again, this is in agreement with the findings of previous studies [66,67]. There was evidence of a linear correlation between a higher BMI and a higher incidence of AKI [68]. The pathophysiology linking obesity and AKI is unclear. One explanation would be that obesity causes a change in renal hemodynamics that may lead to vulnerability to kidney injury [69]. Furthermore, inflammatory cytokine production from adipose tissue during acute illness has been shown to participate in the development of AKI [70]. Subsequently, the increase of intra-abdominal pressure and central venous pressure from obesity was proposed as another mechanism which increases AKI risk [71]. Lastly, the meta-analysis study of COVID-19 suggested that obesity increases severe clinical course, ICU admission, and death among COVID-19 patients [72]. 

The limitations of this study were as follows. First, we excluded around 50% of screened medical records because of the exclusion criteria, such as no SCr investigation or an unconfirmed diagnosis of TAFI. Second, the diagnosis of AKI was based only on SCr criteria.

## 5. Conclusions

The overall prevalence of TAFI-associated AKI was 6.8%. TAFI-associated AKI can lead to an increased mortality rate. The risk factors for TAFI-associated AKI were being male, having respiratory failure or hyperbilirubinemia, and being obese. The combination of these identified risk factors produced a predictive algorithm, which achieves a value of sensitivity (70%) and specificity (72%) similar to previous studies [73]. We suggest that clinicians investigate kidney function in patients with TAFI who also have these associated risk factors to assist in the detection of AKI in its early stages and to offer appropriate management.

## Figures and Tables

**Table 1:** Prevalence of acute kidney injury (AKI) among cases of tropical acute febrile illness (TAFI).

TAFI	Total Cases *n =* 1019 *n*	Non-AKI Cases *n =* 950 *n* (%)	AKI Cases *n =* 69 *n* (%)
Dengue (%)	767	726 (94.7)	41 (5.3)
Non-severe (93.7%)	719	693 (96.3)	26 (3.6)
Severe (6.3%)	48	33 (4.5)	15 (31.3)
Malaria (%)	131	117 (89.3)	14 (10.7)
Pf (28.2)	37	27 (73.0)	10 (27.0)
Pv (71.8)	94	90 (95.7)	4 (4.3)
Influenza (%)	106	95 (89.6)	11 (10.4)
Influenza A (77.4)	82	73 (89.0)	9 (11.0)
Influenza B (22.6)	24	22 (91.7)	2 (8.3)

**Table 2:** Acute kidney injury (AKI) criteria and staging.

AKI Criteria and Staging			
Conventional Criteria 41/1019 (4%)	RIFLE 42/1019 (4.1%)	AKIN 61/1019 (5.9%)	KDIGO 69/1019 (6.8%)
	Risk: 18 (42.9%)	Stage 1: 36 (59%)	Stage 1: 45 (65.2%)
	Injury: 9 (21.4%)	Stage 2: 9 (14.8%)	Stage 2: 8 (11.6%)
	Failure: 15 (35.7%)	Stage 3: 16 (26.2%)	Stage 3: 16 (23.2%)

**Table 3:** Demographic and clinical characteristics for patients with TAFI with and without AKI.

Parameter	Total Cases *n =* 1019	Non-AKI Group *n =* 950	AKI Group *n =* 69	*p*-Value
Age, median (IQR)	33 (24–48)	32 (24–48)	37 (27–56)	0.01
Age group, *n* (%)				
18–39 years	650 (63.8)	611 (64.3)	39 (56.5)	0.06
41–59 years	240 (23.6)	225 (23.7)	15 (21.7)	
≥60 years	129 (12.7)	114 (12)	15 (21.7)	
Sex, *n* (%)				
Male	565 (55.4)	515 (54.2)	50 (72.5)	0.003
Female	454 (44.6)	435 (45.8)	19 (27.5)	
BMI, median (IQR)	23.1 (20.4–26.7)	23 (20.5–26.4)	25.7 (22.7–30.8)	<0.001
Body temperature (°C), median (IQR)	38.2 (37.8–39)	38.2(37.8–39)	38.5 (38–39.3)	0.009
Underlying disease, *n* (%)				
Diabetes mellitus	83 (8.1)	72 (7.6)	11 (15.9)	0.01
Hypertension	141 (13.8)	127 (13.4)	14 (20.3)	0.1
Dyslipidaemia	81 (7.9)	73 (7.7)	8 (11.6)	0.2
Other underlying diseases *	141 (13.8)	120 (12.6)	21(30.4)	<0.001
Dyspnea, *n* (%)	35 (3.4%)	25(2.6%)	10(14.5%)	<0.001
Nausea and vomiting, *n* (%)	378 (37.1)	359 (37.8)	19 (27.5)	0.8
High-grade fever, *n* (%)	191 (18.7)	171 (18)	20 (29)	0.02
Chills, *n* (%)	430(42.2)	401 (42.2)	29(42)	0.9
Headache, *n* (%)	540 (53%)	509 (53.6)	31 (44.9)	0.16
Myalgia, *n* (%)	655 (64.3%)	624 (65.7)	31 (44.9)	0.001
Arthralgia, *n* (%)	43 (4.2)	41 (4.3)	2 (2.9)	0.8
Pallor, *n* (%)	29 (2.8)	22 (2.3)	7 (10.1)	0.002

* Including chronic kidney disease, hepatitis B virus, human immunodeficiency virus, and asthma.

**Table 4:** Laboratory test results among patients with TAFI with and without AKI.

Parameter	Overall		Non-AKI Group		AKI Group		*p*-Value
	*n*	Median (IQR)	*n*	Median (IQR)	*n*	Median (IQR)	
WBC (×10^3^/μL) 10	1019	3.9 (2.8–6.1)	950	3.9 (2.7–6)	69	5.2 (4–8.3)	<0.001
Neutrophils (%)	1019	53 (36–68)	950	52 (35–67)	69	63 (51.5–73)	<0.001
Lymphocytes (%)	1016	24 (15–33)	949	25 (16–34)	69	16 (10–25)	<0.001
Atypical lymphocytes (%)	936	9 (4–18)	872	9 (4–18)	64	5 (2–10.8)	<0.001
RBC (×10^6^/μL)	1019	4.9 (4.9–5.4)	950	4.9 (4.5–5.5)	69	4.7 (4.1–5.4)	0.01
Platelets (×10^3^/μL) /µL)	1019	71 (39–124)	951	72 (39.8–125)	69	55 (29–112)	0.079
Hemoglobin (g/dL)	1019	13.7 (12.5–15)	951	13.7 (12.6–15)	69	13.7 (10.9–15.3)	0.35
Hematocrit (%)	1019	40.6 (37.5–44)	951	40.6 (37.5–43.9)	69	40.4 (33.2–44.7)	0.3
Serum creatinine (mg/dL)	1019	0.84 (0.67–1.02)	950	0.82 (0.66–0.99)	69	1.3 (1.09–1.8)	N/A
BUN (mg/dL)	992	9.9 (7.5–13)	926	9.6 (7.4–12.4)	66	17.2 (12.6–24.8)	N/A
Serum Na (mmol/L)	910	137 (134–139)	845	137 (134–139)	65	135 (131–138)	0.002
Serum K (mmol/L)	910	3.7 (3.5–4)	845	3.7 (3.5–4)	65	4 (3.7–4.3)	<0.001
Serum HCO^3^ (mmol/L)	910	24 (22–26)	845	24 (22–26)	65	22 (18.5–24)	<0.001
Serum albumin (g/L)	637	4 (3.6–4.2)	579	4 (3.6–4.2)	58	3.6 (2.8–4.1)	0.001
Serum total bilirubin (mg/dL)	637	0.6 (0.4–1.1)	579	0.5 (0.4–1)	58	1.5 (0.7–3.2)	<0.001
AST (IU/L)	878	87 (45–180.3)	822	86 (44–175.3)	56	111 (58–360)	0.015
ALT (IU/L)	879	58 (32–121)	823	58 (32–117)	56	69 (35–209)	0.11

**Table 5:** Complications and outcomes among patients with TAFI with and without AKI.

Parameters	Overall Cases *n* = 1019 *n* (%)	Non-AKI Group *n* = 950 *n* (%)	AKI Group *n* = 69 *n* (%)	*p*-Value
Leukocytosis	56 (5.5)	44 (4.6)	12 (17.4)	<0.001
Severe thrombocytopenia	333 (32.7)	304 (32)	29 (42)	0.086
Severe transaminitis (*n* = 882)	69 (7.8)	56 (6.8)	13 (23.2)	<0.001
Hypoalbuminemia (*n* = 637)	111 (17.4)	90 (15.5)	21 (36.2)	<0.001
Metabolic acidosis (*n* = 909)	11 (1.2)	1 (0.1)	10 (15.4)	<0.001
Respiratory failure	62 (6.1)	39 (4.1)	23 (33.3)	<0.001
Multi-organ dysfunction	18 (1.8)	5 (0.5)	13 (18.8)	<0.001
Intensive care unit	43 (4.2)	23 (2.4)	20 (29)	<0.001
Inotropic drug use	13 (1.3)	0 (0)	13 (18.8)	<0.001
Dialysis	14 (1.4)	0 (0)	14 (20.3)	<0.001
Hospital stay > 3 days	507 (49.8)	451 (47.5)	56 (81.2)	0.02
Death	7 (0.68)	0 (0)	7 (10.1)	<0.0001

**Table 6:** Factors associated with AKI by univariate and multivariate analysis.

Variable	Univariate Analysis		Multivariate Analysis	
	*p*-Value	OR (95% CI)	*p*-Value	AOR (95% CI)
Age > 40	0.1	1.49 (0.91–2.4)		1.8 (0.8–3.8)
Male	0.003	2.2 (1.3–3.8)	0.01	3.1 (1.3–7.4)
Diabetes mellitus	0.01	2.3 (1.2–4.6)	-	0.7 (0.2–2.4)
Respiratory failure	<0.001	11.7 (6.4–21.2)	0.008	4.6 (1.5–14.1)
Hypoalbuminemia	<0.001	0.3 (0.1–0.5)		1.5 (0.6–3.5)
Hyperbilirubinemia	<0.001	4.5 (2.6–7.9)	0.02	2.4 (1.1–4.9)
Severe transaminitis	<0.001	4.2 (2.2–8.3)	-	2.3 (0.8–6.5)
Obesity	<0.001	2.9 (1.6–5.2)	0.003	2.9 (1.4–6)

AOR, adjusted odds ratio; CI, confidence interval.

## Data Availability

Due to the patients’ confidentiality, data sharing is not applicable to this article.

## Supplementary Materials

The following supporting information can be downloaded at: https://www.mdpi.com/article/10.3390/tropicalmed8030147/s1, File S1: Supplementary Materials of Clinical characteristics of acute kidney injury associated with tropical acute febrile illness. References [74,75,76,77] are cited in the supplementary materials.
