# Supplementary material for: Clinical Characteristics of Acute Kidney Injury Associated with Tropical Acute Febrile Illness
Source: Trop Med Infect Dis. 2023 Feb 27;8(3):147. doi: 10.3390/tropicalmed8030147 (PMC10056292; doi:10.3390/tropicalmed8030147)
Supplement: Supplementary file 1 [file tropicalmed-08-00147-s001.zip › tropicalmed-2141843-SI.pdf]

Tropical Medicine and Infectious Disease

MDPI

Supplementary Materials of Clinical characteristics of acute kidney injury associated with tropical acute febrile illness

A. Definitions of AKI.

Criteria name	Definition
Conventional [1]	Percentage increase in SCr of ≥ 50% to a final value of SCr >1.5 mg/dL (133 µmol/L).
RIFLE (Risk, Injury, Failure, Loss of kidney function, and End-stage kidney disease) [2]	An increase in SCr to ≥1.5-times baseline within 7 days; risk, SCr increase 1.5–1.9-times baseline; injury, SCr increase 2.0–2.9-times baseline; failure, SCr increase ≥3.0–times baseline or an increase in SCr ≥4.0 mg/dL; loss, loss of kidney function >4weeks; ESKD (end-stage kidney disease), loss of kidney function >3 months.
AKIN (Acute Kidney Injury Network) [3]	An increase in SCr of ≥0.3 mg/dL within 48 hours; stage 1, SCr increase ≥ 1.5-times baseline; stage 2, SCr increase 2.0–2.9-times baseline; stage 3, SCr increase 3.0-times baseline or an increase in SCr ≥4.0 mg/dL.
KDIGO (Kidney Disease: Improving Global Outcomes) [4]	An increase in SCr of ≥0.3 mg/dL within 48 hours or an increase of ≥1.5 times baseline within 7 days; stage 1, SCr increase >0.3 mg/dL or SCr increase 1.5–1.9-times baseline; stage 2, SCr increase 2–2.9-times baseline; stage 3, SCr increase 3-times baseline, or initiation of renal replacement therapy (RRT)

B. Definition of clinical conditions.

Clinical conditions	Definition
Hyperbilirubinemia	A total serum bilirubin concentration of >1.2 mg/dL.
Hypoalbuminemia	A serum albumin concentration <3.5 g/dL.
Transaminitis	An increase in aspartate aminotransferase (AST) and/or alanine aminotransferase (ALT). This can be further classified into mild transaminitis (elevated AST or ALT of <3-times the upper normal limit),moderate transaminitis (elevated AST or ALT of 3- to 10-times the upper normal limit), and severe transaminitis (AST or ALT of >10-times the upper normal limit)
Metabolic acidosis	A serum bicarbonate concentration <15 mmol/L.
Proteinuria	Protein urine >30 mg/d/L or urine dipstick urine proteinuria +1 or above
Hematuria	A condition at which ≥5 red blood cells (RBC)/mm^3 or RBC >6–10 cells/high-power field (HPF), or dipstick urine blood +2 or above.
Pyuria	The presence of ≥10 white blood cells (WBC)/mm^3, or WBC ≥ 3 cells/HPF, or urine dipstick positive for leukocyte esterase
Severe thrombocytopenia	Thrombocyte count <50,000 cells/mm^3

Respiratory failure	A respiratory rate >35/minute or use of a mechanical ventilator
Multi-organ dysfunction (MOD)	An occurrence of more than one organ failure
Obesity	A body mass index (BMI) of >27.5 kg/m^2

References

1. Angeli, P.; Ginès, P.; Wong, F.; Bernardi, M.; Boyer, T.D.; Gerbes, A.; Moreau, R.; Jalan, R.; Sarin, S.K.; Piano, S.; et al. Diagnosis and management of acute kidney injury in patients with cirrhosis: revised consensus recommendations of the International Club of Ascites. *J Hepatol.* **2015**, *62*, 968–974.
2. Bellomo, R.; Ronco, C.; Kellum, J.A.; Mehta, R.L.; Palevsky, P.; ADQI workgroup. Acute renal failure-definition, outcome measures, animal models, fluid therapy and information technology needs: the Second International Consensus Conference of the Acute Dialysis Quality Initiative (ADQI) Group. *Crit Care* **2004**; *8*, 1–9.
3. Mehta, R.L.; Kellum, J.A.; Shah, S.V.; Molitoris, B.A.; Ronco, C.; Warnock, D.G.; Levin, A.; Acute Kindey Injury Network. Acute Kidney Injury Network: report of an initiative to improve outcomes in acute kidney injury. *Crit Care 2007; 11*, 1–8.
4. Kidney Disease: Improving Global Outcomes (KDIGO). Acute Kidney Injury Work Group. KDIGO Clinical Practice Guidelines for Acute Kidney Injury. *Kidney Int Suppl* **2012**, *2,* 1–138.

Disclaimer/Publisher's Note: The statements, opinions and data contained in all publications are solely those of the individual author(s) and contributor(s) and not of MDPI and/or the editor(s). MDPI and/or the editor(s) disclaim responsibility for any injury to people or property resulting from any ideas, methods, instructions or products referred to in the content.
